# On the Uncertainty Identification for Linear Dynamic Systems Using Stochastic Embedding Approach with Gaussian Mixture Models

**DOI:** 10.3390/s21113837

**Published:** 2021-06-01

**Authors:** Rafael Orellana, Rodrigo Carvajal, Pedro Escárate, Juan C. Agüero

**Affiliations:** 1 Departamento Electrónica, Universidad Técnica Federico Santa María (UTFSM), Av. España 1680, Valparaíso 2390123, Chile; rodrigo.carvajalg@usm.cl (R.C.); juan.aguero@usm.cl (J.C.A.); 2 Advanced Center for Electrical and Electronic Engineering, AC3E, Av. Matta 222, Valparaíso 2580129, Chile; 3 Departamento de Ingeniería Electrica, Facultad de Ingeniería, Universidad de Los Andes, Av. Alberto Carnevalli, Mérida 5101, Venezuela; 4 Instituto de Electricidad y Electrónica, Facultad de Ciencias de la Ingeniería, Universidad Austral de Chile (UACH), Genaral Lagos 2086, Valdivia 5111187, Chile; pedro.escarate@uach.cl

**Keywords:** expectation-maximization, gaussian mixture model, maximum likelihood, stochastic embedding, uncertainty modeling

## Abstract

In control and monitoring of manufacturing processes, it is key to understand model uncertainty in order to achieve the required levels of consistency, quality, and economy, among others. In aerospace applications, models need to be very precise and able to describe the entire dynamics of an aircraft. In addition, the complexity of modern real systems has turned deterministic models impractical, since they cannot adequately represent the behavior of disturbances in sensors and actuators, and tool and machine wear, to name a few. Thus, it is necessary to deal with model uncertainties in the dynamics of the plant by incorporating a stochastic behavior. These uncertainties could also affect the effectiveness of fault diagnosis methodologies used to increment the safety and reliability in real-world systems. Determining suitable dynamic system models of real processes is essential to obtain effective process control strategies and accurate fault detection and diagnosis methodologies that deliver good performance. In this paper, a maximum likelihood estimation algorithm for the uncertainty modeling in linear dynamic systems is developed utilizing a stochastic embedding approach. In this approach, system uncertainties are accounted for as a stochastic error term in a transfer function. In this paper, we model the error-model probability density function as a finite Gaussian mixture model. For the estimation of the nominal model and the probability density function of the parameters of the error-model, we develop an iterative algorithm based on the Expectation-Maximization algorithm using the data from independent experiments. The benefits of our proposal are illustrated via numerical simulations.

## 1. Introduction

System identification is a scientific discipline that studies techniques for modeling and estimating dynamic systems from experimental data, i.e., a number of experiments are performed on the system, and a model is then fitted to the measured data by estimating the corresponding system model parameters [1,2]. Most system identification techniques available in the literature assume that the system model that will be fitted lies in the model set [3]. However, real processes have an arbitrary complexity and using a complex model structure can lead to large variance estimation errors [4]. Moreover, the experimental data is typically obtained with the presence of sensors errors and measurement noise, modeling errors, and external disturbances that can impact on the system model accuracy; see, e.g., Reference [5]. This scenario has been considered for sensor or actuator failure diagnosis and isolation in systems with structural uncertainties and noisy measurements, utilizing residual analysis and Bayesian frameworks to develop failure detection procedures [6,7,8,9].

**Figure 1:**
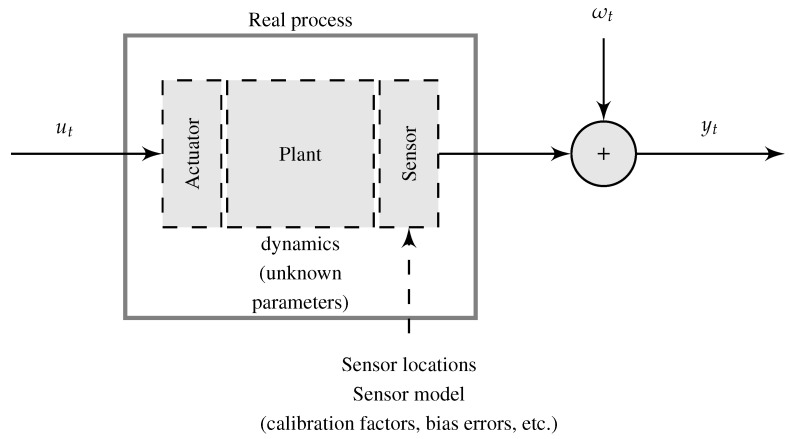
Representation of a real dynamic system with data measurements for system identification.

In Figure 1, a typical representation of a real process and measured experimental data is shown, where $u_{t}$ is the input measurements, $y_{t}$ denotes the output measurements, and $\omega_{t}$ denotes a measurement noise. The real process (*true* system) is represented by the interaction of three components: (i) an actuator, (ii) a plant, and (iii) a sensor. This representation has been used to develop system identification methodologies for flight vehicle development in order to obtain accurate models and to validate mathematical models of the flight vehicle [10]. These system models are required, for example, to perform fault-diagnosis and adaptive control, to analyze handling qualities specification compliance, to develop high-fidelity aerodynamic databases for flight simulators, among others [11]. However, the quality of these results can be affected by the uncertainties incorporated by the (unknown) dynamics that are not modeled, the instrumentation, model simplifications, and measurement noise and sensor errors [12].

On the other hand, in Reference [13], the modeling and parameter identification in structural health monitoring was addressed. The authors considered that the multi-sensor data in a structural health monitoring can provide incomplete information due to multi-sensor faults or errors and then quantified its corresponding uncertainty with a Bayesian framework. Similarly, in Reference [14], a sensor-orientated approach for space situational awareness and traffic management uncertainty quantification was presented, analyzing the implications of the sensor uncertainties in the performance of this systems. A mathematical formulation based on the least square method was developed, providing a methodology to represent the navigation and tracking errors as an uncertainty volume that accurately depicts the size and orientation in space situational awareness evolution.

For these reasons, a suitable formulation of a model that incorporates uncertainties is an important aspect to consider in system identification methods, in order to obtain system models that represent as closely as possible the real process behavior [1,2,10]. Because of its valuable statistical properties [1,2,3], many identification algorithms involve the maximum likelihood (ML) principle with particular system models, such as dynamic systems [15,16,17], static systems [18], systems with quantized output data [19,20,21,22], and communications [23,24], to mention a few. In particular, the ML estimators are asymptotically unbiased. That is, when the number of measurements is not too large, the estimates can be far from the *true* value. This bias is typically accounted for by the measurement noise variance. However, there are scenarios in which the number of sampled-data is, in theory, large enough, and the estimates considerably change from one experiment to another, i.e., there is an uncertainty in the system model that cannot be accounted for by the noise measurements and a large variance.

**Figure 2:**
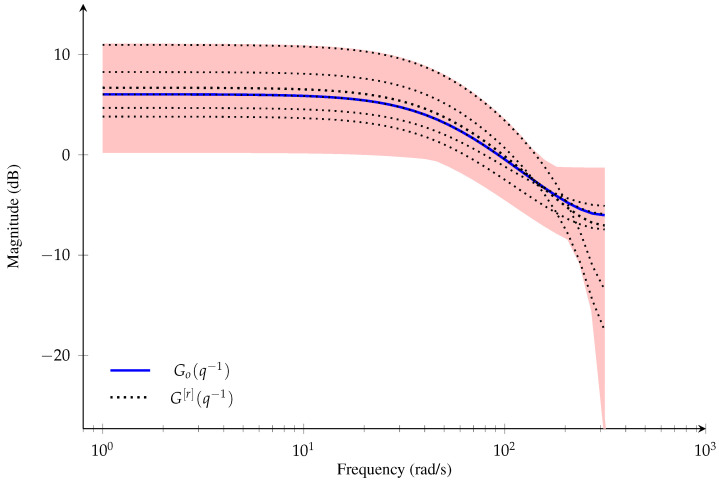
Magnitude of the frequency response for the linear dynamic system in Equation (1).

To illustrate the stochastic embedding approach, the magnitude of the frequency response of a linear dynamic system (assumed to be the *true* system) is shown in Figure 2, where $G^{\left[r\right]}\left(q^{-1}\right)$ is given by

(1) $$G^{\left[r\right]}\left(q^{-1}\right)=G_{o}\left(q^{-1}\right)+G_{o}\left(q^{-1}\right)G_{\Delta }^{\left[r\right]}\left(q^{-1}\right),$$

where $q^{-1}$ denotes the backward shift operator ($q^{-1}x_{t}=x_{t-1}$), *r* denotes a realization of the *true* system, $G_{o}\left(q^{-1}\right)$ is a fixed nominal model, and $G_{\Delta }^{\left[r\right]}\left(q^{-1}\right)$ denotes the *r*th realization of a relative error-model for the *true* system. The red-shaded region represents the area in which possible *true* systems, $G^{\left[r\right]}\left(q^{-1}\right)$, i.e, realizations of the *true* system (represented by black dotted lines), can lie. The error-model can introduce model parameter uncertainties at low frequency range and model structure uncertainties at high frequency range [10]. This system model behavior can be encountered, for instance, in an aeroservoelastic system model (see, e.g., References [5,25]). Aeroservoelastic systems include dynamic coupling due to structural, control, sensor and actuator dynamics that cover both the low frequency and high frequency range. This means that, in an ML framework, the estimates can change considerable from one experiment to another, even if the number of measurements used to obtain an ML estimation is large, due to the structural and parametric uncertainties in the *true* system model. Hence, it is desirable that the models obtained from system identification techniques provide a quantification of their uncertainty, i.e., considering that the uncertainty modeling is part of the structure and estimating a nominal model with an error-model [26,27,28]. This approach of uncertainty modeling can be used for robust control design in which optimization methods can be used to obtain controllers in order to minimize the expected variation (variance) of the *true* system performance from a given desired behavior [29], or to obtain a probabilistic solution for robust control design [30,31].

This alternative view of modeling error-model has been addressed in different frameworks. In Reference [32], the set membership approach was used to deal with the problem of uncertainty modeling in dynamic systems. The authors of Reference [32] considered the error-model estimation in a deterministic framework in which it is unknown-but-bounded and obtaining a set of possible solutions. However, this method does not guarantee a small set of solutions to properly describe the system model uncertainty. In Reference [33], classical prediction error methods (PEM) [2] were used to obtain an estimation of the nominal model. Then, the unmodeled dynamics were estimated from the residuals dynamics using PEM and obtaining the corresponding model error modeling (MEM). Nevertheless, this methodology assumes that the nominal model is available. In References [26,34], a stochastic embedding (SE) approach was used to describe the model uncertainty by considering the model as a realization drawn from an underlying probability space, where the parameters that define the error-model are characterized by a probability density function (PDF). However, a flexible error-model distribution is needed to obtain a correct description of the system model uncertainty. In this approach, the uncertainty can be quantified by using ML estimation considering the parameters of the error-model as hidden variables [4,35] and solving the associated estimation problem with an expectation-maximization (EM) [36] based algorithm under Gaussian assumptions for the error-model distribution. References [4,37] also adopted a Bayesian perspective, where the parameters that define both the nominal model and the error-model can be modeled as realizations of random variables with certain prior distributions, and the posterior densities of the parameters can be estimated. There are works closely related with this framework [38,39] based on the kernel approach for system identification. Under this Bayesian framework, it is possible to obtain an unified system model of both the nominal model and error-model for the system in Equation (1) if all system models are considered finite impulse response (FIR) systems. However, this approach does not deliver the nominal model and the error-model separately when more complex model structures are involved in the *true* system.

In this paper, we propose an ML identification algorithm for modeling the uncertainty in a general class of linear dynamic systems using SE approach. That is, we aim at obtaining an estimation of the nominal model and the red-shaded region in Figure 2. We also consider that the error-model distribution is given by a Gaussian mixture model (GMM). GMMs have been utilized in non-linear filtering [40,41,42], identification of dynamic systems with ML framework [43,44] and a Bayesian framework [45,46,47]. Modeling error-model using SE approach for FIR systems have also been used with Gaussian mixtures distributions [48]. Moreover, GMMs have been used to approximate non-Gaussian distributions in the ML framework (see, e.g., References [17,47,49]) based on the Wiener approximation theorem which established that any PDF with compact support can be approximated by a finite sum of Gaussian distributions (Reference [50], Theorem 3). Combining the SE approach with Gaussian mixture distributions provides a flexible scenario for uncertainty modeling when the error-model distribution does not correspond to a GMM but can be approximated by one; the latter corresponds to the Wiener approximation theorem. The contributions of this paper can be summarized as follows:

(i) We obtain the likelihood function for a linear dynamic system modeled with SE approach and a finite Gaussian mixture distribution. The likelihood function is computed by marginalizing the vector of parameters of the error-model as a hidden variable.
(ii) We propose an EM algorithm to solve the associated ML estimation problem with GMMs, obtaining the estimates of the vector of parameters that define the nominal model and closed-form expressions for the GMM estimators of the error-model distribution.

The remainder of the paper is as follows: In Section 2, the system of interest for uncertainty modeling with SE framework is stated. In Section 3, the estimation problem for uncertainty modeling in a linear dynamic system is addressed using SE approach with GMMs. In Section 4, an EM algorithm is presented to solve the estimation problem. Numerical simulation examples are presented in Section 5. Finally, in Section 6, we present our conclusions.

## 2. Uncertainty Modeling for Linear Dynamic Systems Using Stochastic Embedding Approach

### 2.1. System Description

The system of interest in this paper is as follows:

(2) $$y_{t}^{\left[r\right]}=G^{\left[r\right]}\left(q^{-1}\right)u_{t}^{\left[r\right]}+H\left(q^{-1},\theta \right)\omega_{t}^{\left[r\right]},$$

where r=1,…,M denotes the *r*-th realization of the system, *M* corresponds the number of independent experiments or batches, t=1,…,N denotes the *t*-th measurement, *N* is the data length, $y_{t}^{\left[r\right]}$ is the output signal, $u_{t}^{\left[r\right]}$ denotes the input signal, $\omega_{t}^{\left[r\right]}$ is a zero-mean Gaussian white noise with variance $\sigma^{2}$, and $H(q^{-1},\theta )$ is the noise model parametrized by θ. We consider that the system $G^{\left[r\right]}\left(q^{-1}\right)$ can be described as follows (see, e.g., References [26,37]):

(3) $$G^{\left[r\right]}\left(q^{-1}\right)=G_{o}\left(q^{-1},\theta \right)\left(1+G_{\Delta }\left(q^{-1},\eta^{\left[r\right]}\right)\right),$$

where $G_{o}\left(q^{-1},\theta \right)$ is the nominal system model parametrized by θ, and $G_{\Delta }\left(q^{-1},\eta^{\left[r\right]}\right)$ is the error-model parametrized by $\eta^{\left[r\right]}$. Here, we consider that the PDF of the error-model in Equation (2) is a finite Gaussian mixture distribution given by:

(4) $$p\left(\eta^{\left[r\right]}|\gamma \right)=\sum_{i=1}^{\kappa }\alpha_{i}N\left(\eta^{\left[r\right]};\mu_{i},\Gamma_{i}\right),$$

(5) $$\gamma ={\left[\underset{\gamma_{1}}{\underset{︸}{\alpha_{1}\;\mu_{1}\;\Gamma_{1}}}\;\cdots \;\underset{\gamma_{\kappa }}{\underset{︸}{\alpha_{\kappa }\;\mu_{\kappa }\;\Gamma_{\kappa }}}\right]}^{T},$$

where ${\left[\cdot \right]}^{T}$ denotes the transpose operator, $\alpha_{i}>0$, $\sum_{i=1}^{\kappa }\alpha_{i}=1$, and $N(\eta^{\left[r\right]};\mu_{i},\Gamma_{i})$ represents a Gaussian PDF with mean value $\mu_{i}$ and covariance matrix $\Gamma_{i}$. For simplicity of the presentation, we consider that $H(q^{-1},\theta )$ in Equation (2) is part of the nominal model, i.e., it is only parametrized by θ. The nominal system model in Equation (3) is given by

(6) $$G_{o}\left(q^{-1},\theta \right)=\frac{B(q^{-1},\theta )}{A(q^{-1},\theta )},$$

where

(7) $$B\left(q^{-1},\theta \right)=b_{1}q^{-1}+\cdots +b_{n_{b}}q^{-nb},$$

(8) $$A\left(q^{-1},\theta \right)=1+a_{1}q^{-1}+\cdots +a_{n_{a}}q^{-na}.$$

Similarly, the noise system model in Equation (2) is given by

(9) $$H\left(q^{-1},\theta \right)=\frac{C(q^{-1},\theta )}{D(q^{-1},\theta )},$$

with

(10) $$C\left(q^{-1},\theta \right)=1+c_{1}q^{-1}+\cdots +c_{n_{c}}q^{-nc},$$

(11) $$D\left(q^{-1},\theta \right)=1+d_{1}q^{-1}+\cdots +d_{n_{d}}q^{-nd}.$$

Notice that we use the term nominal model to refer to the system model $G_{o}\left(q^{-1},\theta \right)$ and the noise system model $H(q^{-1},\theta )$. We also consider that the error-model $G_{\Delta }\left(q^{-1},\eta^{\left[r\right]}\right)$ in Equation (3) is a linear regression as follows:

(12) $$G_{\Delta }\left(q^{-1},\eta^{\left[r\right]}\right)=\eta_{0}^{\left[r\right]}+\eta_{1}^{\left[r\right]}q^{-1}+\cdots +\eta_{n_{\Delta }}^{\left[r\right]}q^{-n_{\Delta }},$$

where $\eta^{\left[r\right]}\in R^{n_{\Delta }\times 1}$.

### 2.2. Standing Assumptions

The problem of interest is to estimate the vector of parameters, $\beta ={\left[\theta^{T}\;\gamma^{T}\;\sigma^{2}\right]}^{T}$, that defines the parameters of the nominal model, error-model and the noise variance. In addition, we consider that $\beta_{0}$ is the *true* vector of parameters that defines the *true* model. In order to formulate the ML estimation algorithm, we introduce the following standing assumptions:

**Assumption** **1.** *The system in Equation (2) is operating in open loop, and the input signal $u_{t}^{\left[r\right]}$ is an exogenous deterministic signal for each r independent experiment. In addition, the data of M independent experiments are available.*

**Assumption** **2.** *The nominal model does not change from one experiment to another, whilst the error-model $G_{\Delta }\left(q^{-1},\eta^{\left[r\right]}\right)$ may change for each experiment, and all the realizations of $\eta ={\left\{\eta^{\left[r\right]}\right\}}_{r=1}^{M}$ are drawn from the same PDF parametrized by γ.*

**Assumption** **3.** *The vector of parameters $\beta_{0}$, the input signal $u_{t}^{\left[r\right]}$, and the noise $\omega_{t}^{\left[r\right]}$ in Equation (2) satisfy regularity conditions, guaranteeing that the ML estimate $\beta_{ML}$ converges (in probability or a.s.) to the true solution $\beta_{0}$ as N→∞.*

**Assumption** **4.** *The orders $n_{a}$, $n_{b}$, $n_{c}$, $n_{d}$, and $n_{\Delta }$ of the polynomials of system in Equation (2) and the number of components κ of the error-model distribution in Equation (4) are known.*

**Assumption** **5.** *The system in Equation (2) is asymptotically stable, and its models $G^{\left[r\right]}\left(q^{-1}\right)$ and $H(q^{-1},\theta )$ have no poles-zeros on the unit circle and have no pole-zero cancellations. The noise system model $H(q^{-1},\theta )$ is also a minimum-phase system.*

Assumption 2 is needed to develop an ML methodology based on the uncertainty modeling with SE approach. Assumption 3 is necessary to develop an estimation algorithm that holds the asymptotic properties of the ML estimator. Assumption 4 can be relaxed by considering a dynamic system model that includes parametric uncertainties with a different error-model structure that we assume in Equation (3). We will address this case in Section 5. Assumption 5 is necessary to obtain an asymptotically unbiased ML estimator [51] and a system model that is controllable [52].

## 3. Maximum Likelihood Estimation for Uncertainty Modeling Using GMMs

In this section, we develop an ML estimation algorithm for system in Equation (2) to obtain both the nominal model and the error-model distribution. We consider that the observed data $Y^{\left[r\right]}=\left[y_{1}^{\left[r\right]}\;\cdots \;y_{N}^{\left[r\right]}\right]$ is a collection of measurements for each experiment. We use capital letters to denote the vector of signals, $u_{t}$ or $\omega_{t}$ for t=1,…,N. Then, the system in Equation (2) can be described as follows:

(13) $$Y^{\left[r\right]}=G\left(\theta \right)U^{\left[r\right]}+\Psi^{\left[r\right]}\left(\theta \right)\eta^{\left[r\right]}+W^{\left[r\right]},$$

where $Y^{\left[r\right]},U^{\left[r\right]},W^{\left[r\right]}\in R^{N\times 1}$, $\theta \in R^{n_{o}\times 1}$, $\eta^{\left[r\right]}\in R^{n_{\Delta }\times 1}$, $G\left(\theta \right)U^{\left[r\right]}\in R^{N\times 1}$, $\Psi^{\left[r\right]}\left(\theta \right)\in R^{N\times n_{\Delta }}$. The term $G\left(\theta \right)U^{\left[r\right]}$ corresponds to the output response corresponding to the nominal model structure G(θ), $\Psi^{\left[r\right]}\left(\theta \right)\eta^{\left[r\right]}$ corresponds to the output signal due to the error-model structure in Equation (3), and $W^{\left[r\right]}∼N\left(0,\sigma^{2}I_{N}\right)$ ($I_{x}$ represents the identity with dimension given by *x*). Notice that G(θ) involves the nominal model structure utilized for $G_{o}\left(q^{-1},\theta \right)$ and $H(q^{-1},\theta )$ in Equation (2). The term $\Psi^{\left[r\right]}\left(\theta \right)$ describes the model structure given by the product $G_{o}\left(q^{-1},\theta \right)G_{\Delta }\left(q^{-1},\eta^{\left[r\right]}\right)$ in Equation (3).

**Remark** **1.** *The system model in Equation (13) consider that the error-model structure $\Psi^{\left[r\right]}\left(\theta \right)\eta^{\left[r\right]}$ is a linear regression, and the nominal model structure G(θ) can have an arbitrary structure [37]. The fact of considering the error-model as a linear regression can provide flexibility, lower computational complexity, and contain FIR model of arbitrary orders, Laguerre and Kautz models (see, e.g., Reference [53]).*

We define the vector of parameters to be estimated as $\beta ={\left[\theta^{T}\;\gamma^{T}\sigma^{2}\right]}^{T}$ in order to formulate the ML estimator for the system in Equation (13):

(14) $$\theta ={\left[b_{1}\;a_{1}\;c_{1}\;d_{1}\;\cdots \;b_{n_{b}}\;a_{n_{a}}\;c_{n_{c}}\;d_{n_{d}}\right]}^{T},$$

where θ is the vector of parameters of the nominal model, γ is the vector of parameters of the error-model distribution as a GMM in Equation (4), and $\sigma^{2}$ is the noise variance. The corresponding ML estimation algorithm using GMMs is obtained as follows:

**Lemma** **1.** *Consider the parameters to be estimated as $\beta ={\left[\theta^{T}\;\gamma^{T}\sigma_{\omega }^{2}\right]}^{T}$ using Equations (5) and (14). Under the standing assumptions and using Equation (13), the ML estimator for the system model in Equation (2) is given by:*

(15) $${\hat{\beta }}_{ML}=\arg\;\max_{\beta }\;\ell \left(\beta \right)\;\;s.t.\;\;\sum_{i=1}^{\kappa }\alpha_{i}=1,\;0\leq \alpha_{i}\leq 1,$$

*where the log-likelihood function is given by*

(16) $$\ell \left(\beta \right)=\sum_{r=1}^{M}\log\left\{\sum_{i=1}^{\kappa }\alpha_{i}N\left(Y^{\left[r\right]};{\bar{\mu }}_{ir},{\bar{\Sigma }}_{ir}\right)\right\},$$

(17) $${\bar{\mu }}_{ir}=\Psi^{\left[r\right]}\left(\theta \right)\mu_{i}+G\left(\theta \right)U^{\left[r\right]},$$

(18) $${\bar{\Sigma }}_{ir}=\sigma^{2}I_{N}+\Psi^{\left[r\right]}\left(\theta \right)\Gamma_{i}{\left[\Psi^{\left[r\right]}\left(\theta \right)\right]}^{T}.$$

**Proof.**  See Appendix A. □

The result obtained from Lemma 1 differs from the classical ML formulation, where the measurement data of one experiment is used to obtain one estimate of the parameters of interest (see, e.g., References [1,2]). In contrast, from Lemma 1, the ML estimator considers the measurements of *M* independent experiments to obtain one estimation of the system model, i.e, the nominal model and error-model distribution as a GMM. Notice that ${\bar{\mu }}_{ir}$ and ${\bar{\Sigma }}_{ir}$ in Equations (17) and (18), respectively, depend on the vector of parameters β.

The optimization problem in Equation (15) is typically solved using gradient-based methods. However, the log-likelihood function in Equation (16) contains a matrix ${\bar{\Sigma }}_{i}^{\left[r\right]}$ with dimension N×N that may seem cumbersome to solve for the optimization problem [37,54]. Moreover, the log-likelihood function involves a logarithm of a summation that depends of the number of components κ of the GMM, and it may be difficult to solve when the number of components in the GMM is high [55]. The EM algorithm [36] can provide a solution to deal with this difficulty since combination of an EM algorithm with GMMs typically provides closed-form estimators for the GMM parameters [56,57].

## 4. An EM Algorithm with GMMs for Uncertainty Modeling in Linear Dynamic Systems

The EM algorithm is a popular tool for identifying linear and non-linear dynamic systems in the time domain (see, e.g., References [58,59]) and frequency domain [60]. In particular, a common strategy used in the formulation of an EM algorithm with GMMs is to consider a hidden (latent) variable, modeled as an indicator, that determines from which GMM component an observation comes from Reference [57]. To solve the estimation problem in Equation (15), an EM algorithm with GMMs is developed from the definition of the likelihood function from the observed data of all experiments $Y^{\left[1:M\right]}=\left[Y^{\left[1\right]},\;\cdots ,Y^{\left[M\right]}\right]$ and a hidden discrete random variable, $\zeta_{1:M}$, i.e., defining the likelihood function in Equation (16) using the complete data, $\left\{Y^{\left[1:M\right]},\zeta_{1:M}\right\}$. This hidden random variable $\zeta_{r}$ is an indicator that determines if observations $Y^{\left[r\right]}$ arise from the *i*th component of the GMM. Hence, the EM algorithm is given by (see, e.g., References [36,57]):

(19) $$Q\left(\beta ,{\hat{\beta }}^{\left(m\right)}\right)=E\left\{\log\left[p\left(Y^{\left[1:M\right]},\zeta_{1:M}|\beta \right)\right]|Y^{\left[1:M\right]},{\hat{\beta }}^{\left(m\right)}\right\},$$

(20) $${\hat{\beta }}^{\left(m+1\right)}=\arg\max_{\beta }\;Q\left(\beta ,{\hat{\beta }}^{\left(m\right)}\right),\;\;s.t.\;\;\sum_{i=1}^{\kappa }\alpha_{i}=1,\;\;0\leq \alpha_{i}\leq 1,$$

where $E\left\{a|b\right\}$ and p(a|b) denote the expected value and the PDF of the random variable *a* given the random variable *b*, respectively, ${\hat{\beta }}^{\left(m\right)}$ is the current estimate, $p(Y,\zeta_{1:M}|\beta )$ is the joint PDF of $Y^{\left[1:M\right]}$ and $\zeta_{1:M}$, and $Q(\beta ,{\hat{\beta }}^{\left(m\right)})$ is the auxiliary function of the EM algorithm. Notice that Equations (19) and (20) correspond to the *E-step* and *M-step* of the EM algorithm, respectively [36].

In order to develop the iterative algorithm in Equations (19) and (20), we obtain the following result. This will be used to compute $Q(\beta ,{\hat{\beta }}^{\left(m\right)})$ in Equation (19):

**Lemma** **2.** *Consider the vector of parameters $\beta ={\left[\theta^{T}\;\gamma^{T}\sigma^{2}\right]}^{T}$ defined in Equations (5) and (14). The joint log-PDF, $\log[p\left(Y^{\left[1:M\right]},\zeta_{1:M}|\beta \right)]$, in Equation (19) is given by*

(21) $$\begin{array}{c} \log\left[p\left(Y^{\left[1:M\right]},\zeta_{1:M}|\beta \right)\right]=\sum_{r=1}^{M}\sum_{i=1}^{\kappa }\log\left[p\left(\zeta_{r}=i|\beta \right)\right]+\log\left[N\left(Y^{\left[r\right]};{\bar{\mu }}_{ir},{\bar{\Sigma }}_{ir}\right)\right], \end{array}$$

*where ${\bar{\mu }}_{ir}$ and ${\bar{\Sigma }}_{ir}$ are given by Equations (17) and (18), respectively.*

**Proof.**  See Appendix B. □

Based on the coordinate descent algorithm [61], we obtain a new estimate in Equation (20) using the following steps:

(1) Fixing the vector of parameters θ at its value from the current iteration ${\hat{\theta }}^{\left(m\right)}$ to optimize Equation (19) with respect to the GMM parameters γ and the noise variance $\sigma^{2}$.
(2) Fixing the GMM parameters and the noise variance at their values from the iteration ${\hat{\gamma }}^{\left(m+1\right)}$ and ${\hat{\sigma }}^{2^{\left(m+1\right)}}$ and to solve the optimization problem in Equation (20) to obtain ${\hat{\theta }}^{\left(m+1\right)}$.

From Lemma 2, our proposed EM algorithm can be computed as follows:

**Theorem** **1.** *Consider the vector of parameters to be estimated $\beta ={\left[\theta^{T}\;\gamma^{T}\sigma^{2}\right]}^{T}$ defined in Equations (5) and (14) for the system in Equation (2). Substituting Equation (21) in Equation (19), the E-step in the EM algorithm is given by*

(22) $$Q\left(\beta ,{\hat{\beta }}^{\left(m\right)}\right)=\sum_{r=1}^{M}\sum_{i=1}^{\kappa }\log\left[\alpha_{i}N\left(Y^{\left[r\right]};{\bar{\mu }}_{ir},{\bar{\Sigma }}_{ir}\right)\right]{\hat{\zeta }}_{ir}^{\left(m\right)},$$

(23) $${\hat{\zeta }}_{ir}^{\left(m\right)}=\frac{{\hat{\alpha }}_{i}^{\left(m\right)}N\left(Y^{\left[r\right]};{\hat{\bar{\mu }}}_{ir}^{\left(m\right)},{\hat{\bar{\Sigma }}}_{ir}^{\left(m\right)}\right)}{\sum_{l=1}^{\kappa }{\hat{\alpha }}_{l}^{\left(m\right)}N\left(Y^{\left[r\right]};{\hat{\bar{\mu }}}_{lr}^{\left(m\right)},{\hat{\bar{\Sigma }}}_{lr}^{\left(m\right)}\right)},$$

*where ${\hat{\bar{\mu }}}_{ir}^{\left(m\right)}$ and ${\hat{\bar{\Sigma }}}_{ir}^{\left(m\right)}$ are given by:*

(24) $${\hat{\bar{\mu }}}_{ir}^{\left(m\right)}=\Psi^{\left[r\right]}\left({\hat{\theta }}^{\left(m\right)}\right){\hat{\mu }}_{i}^{\left(m\right)}+G\left({\hat{\theta }}^{\left(m\right)}\right)U^{\left[r\right]},$$

(25) $${\hat{\bar{\Sigma }}}_{ir}^{\left(m\right)}={\hat{\sigma }}^{2^{\left(m\right)}}I_{N}+\Psi^{\left[r\right]}\left({\hat{\theta }}^{\left(m\right)}\right){\hat{\Gamma }}_{i}^{\left(m\right)}{\left[\Psi^{\left[r\right]}\left({\hat{\theta }}^{\left(m\right)}\right)\right]}^{T}.$$

*Consider the maximization problem stated in Equation (20) using Equation (22). The M-step in the EM algorithm is carried out using the following steps:*

*(i)*  *Solving Equation (20) using Equation (22) with $\theta ={\hat{\theta }}^{\left(m\right)}$:*
  (26) $${\hat{\alpha_{i}}}^{\left(m+1\right)}=\left(\sum_{r=1}^{M}{\hat{\zeta }}_{ir}^{\left(m\right)}\right)/M,$$
  (27) $${\hat{\mu }}_{i}^{\left(m+1\right)}=\left(\sum_{r=1}^{M}{\hat{\tilde{\mu }}}_{ir}^{\left(m\right)}{\hat{\zeta }}_{ir}^{\left(m\right)}\right)/\left(\sum_{r=1}^{M}{\hat{\zeta }}_{ir}^{\left(m\right)}\right),$$
  (28) $$\begin{array}{c} {\hat{\Gamma }}_{i}^{\left(m+1\right)}=\left(\sum_{r=1}^{M}\left[\left({\hat{\tilde{\mu }}}_{ir}^{\left(m\right)}-{\hat{\mu }}_{i}^{\left(m\right)}\right){\left({\hat{\tilde{\mu }}}_{ir}^{\left(m\right)}-{\hat{\mu }}_{i}^{\left(m\right)}\right)}^{T}+{\hat{\tilde{\Sigma }}}_{ir}^{\left(m\right)}\right]{\hat{\zeta }}_{ir}^{\left(m\right)}\right)/\left(\sum_{r=1}^{M}{\hat{\zeta }}_{ir}^{\left(m\right)}\right), \end{array}$$
  (29) $${\hat{\sigma }}^{2^{\left(m+1\right)}}=\left(\sum_{r=1}^{M}\sum_{i=1}^{\kappa }R_{ir}^{\left(m\right)}{\hat{\zeta }}_{ir}^{\left(m\right)}\right)/NM,$$
  *with*
  (30) $${\hat{P}}_{ir}^{\left(m\right)}={\hat{\Gamma }}_{i}^{\left(m\right)}\Psi^{\left[r\right]}{\left({\hat{\theta }}^{\left(m\right)}\right)}^{T}{\left({\hat{\bar{\Sigma }}}_{ir}^{\left(m\right)}\right)}^{-1},$$
  (31) $${\hat{\tilde{\mu }}}_{ir}^{\left(m\right)}={\hat{\mu }}_{i}^{\left(m\right)}+{\hat{P}}_{ir}^{\left(m\right)}\left(Y^{\left[r\right]}-{\hat{\bar{\mu }}}_{ir}^{\left(m\right)}\right),$$
  (32) $${\hat{\tilde{\Sigma }}}_{ir}^{\left(m\right)}=\left(I_{n_{\Delta }}-{\hat{P}}_{ir}^{\left(m\right)}\Psi^{\left[r\right]}\left({\hat{\theta }}^{\left(m\right)}\right)\right){\hat{\Gamma }}_{i}^{\left(m\right)},$$
  (33) $$\begin{array}{cc} R_{ir}^{\left(m\right)} & ={\left(Y^{\left[r\right]}-G\left({\hat{\theta }}^{\left(m\right)}\right)U^{\left[r\right]}-\Psi^{\left[r\right]}\left({\hat{\theta }}^{\left(m\right)}\right){\hat{\tilde{\mu }}}_{ir}^{\left(m\right)}\right)}^{T}\\ \; & \left(Y^{\left[r\right]}-G\left({\hat{\theta }}^{\left(m\right)}\right)U^{\left[r\right]}-\Psi^{\left[r\right]}\left({\hat{\theta }}^{\left(m\right)}\right){\hat{\tilde{\mu }}}_{ir}^{\left(m\right)}\right)+\mathrm{tr}\left(\Psi^{\left[r\right]}{\left({\hat{\theta }}^{\left(m\right)}\right)}^{T}{\hat{\tilde{\Sigma }}}_{ir}^{\left(m\right)}\Psi^{\left[r\right]}\left({\hat{\theta }}^{\left(m\right)}\right)\right), \end{array}$$
  *where tr(·) denotes the trace operator.*
*(ii)*  *Solving Equation (20) using Equation (22) with $\gamma ={\hat{\gamma }}^{\left(m+1\right)}$:*
  (34) $${\hat{\theta }}^{\left(m+1\right)}=\arg\min_{\theta }\sum_{r=1}^{M}\sum_{i=1}^{\kappa }{\hat{\zeta }}_{ir}^{\left(m\right)}B_{ir}\left(\theta ,\theta^{\left(m\right)}\right),$$
  *where*
  (35) $$\begin{array}{cc} B_{ir}\left(\theta ,\theta^{\left(m\right)}\right) & ={\left(Y^{\left[r\right]}-G\left(\theta \right)U^{\left[r\right]}-\Psi^{\left[r\right]}\left(\theta \right){\hat{\tilde{\mu }}}_{ir}^{\left(m+1\right)}\right)}^{T}\\ \; & \left(Y^{\left[r\right]}-G\left(\theta \right)U^{\left[r\right]}-\Psi^{\left[r\right]}\left(\theta \right){\hat{\tilde{\mu }}}_{ir}^{\left(m+1\right)}\right)+\mathrm{tr}\left(\Psi^{\left[r\right]}{\left(\theta \right)}^{T}{\hat{\tilde{\Sigma }}}_{ir}^{\left(m+1\right)}\Psi^{\left[r\right]}\left(\theta \right)\right). \end{array}$$

**Proof.**  See Appendix C. □

From Theorem 1, we obtain closed-form expressions for the GMM and noise variance estimators. They are computed from the definition of the auxiliary function $Q(\beta ,{\hat{\beta }}^{\left(m\right)})$ using the complete log-likelihood function in Equation (21). That differs from the procedure proposed in Reference [49], where an auxiliary function is built without explicitly defining a hidden variable. However, the auxiliary function obtained in Reference [49] is equal to Equation (19) and the estimators are obtained from Equations (26)–(29) and Equation (34).

Finally, our estimation algorithm is summarized in Algorithm 1.
**Algorithm 1** (EM algorithm with GMMs).     **Input**$Y^{\left[1:M\right]}$, $U^{\left[1:M\right]}$, *M*, κ, ${\hat{\theta }}^{\left(0\right)}$, ${\hat{\gamma }}^{\left(0\right)}$ and ${\hat{\sigma }}^{2^{\left(0\right)}}$.     **Output**$\hat{\theta }$, $\hat{\gamma }$ and ${\hat{\sigma }}^{2}$. 1:m←0 2:**procedure**E-step 3:    Compute ${\hat{\bar{\mu }}}_{ir}^{\left(m\right)}$ and ${\hat{\bar{\Sigma }}}_{ir}^{\left(m\right)}$ from Equations (24) and (25). 4:    Compute ${\hat{\zeta }}_{ir}^{\left(m\right)}$ from Equation (23). 5:    Compute ${\hat{\tilde{\mu }}}_{ir}^{\left(m\right)}$ and ${\hat{\tilde{\Sigma }}}_{ir}^{\left(m\right)}$ from Equations (31) and (32). 6:**end procedure** 7:**procedure**M-step 8:    Estimate ${\hat{\gamma }}^{\left(m+1\right)}$, ${\hat{\sigma }}^{2^{\left(m+1\right)}}$ from Equations (26)–(29). 9:    Compute $B_{ir}\left(\theta ,\theta^{\left(m\right)}\right)$ from Equation (35) using ${\hat{\gamma }}^{\left(m+1\right)}$ and ${\hat{\theta }}^{\left(m\right)}$.10:    Estimate ${\hat{\theta }}^{\left(m+1\right)}$ by solving Equation (34).11:**end procedure**12:**if** stopping criterion is not satisfied **then**13:    m←m+1, **return** to 214:**else**15:    $\hat{\theta }\leftarrow {\hat{\theta }}^{\left(m+1\right)}$, $\hat{\gamma }\leftarrow {\hat{\gamma }}^{\left(m+1\right)}$, ${\hat{\sigma }}^{2}\leftarrow {\hat{\sigma }}^{2^{\left(m+1\right)}}$16:**end if**17:End

## 5. Numerical Examples

In this section, we present two numerical examples with different approaches to illustrate the benefits of our proposal for modeling both the nominal model and the error-model in the system of Equation (2). This approach of numerical simulations is often used when new estimation algorithms are tested in order to evaluate the estimation performance in conditions that could reduce the safety levels or deal with problems for which experiments cannot be planned [62,63].

In the first example, we consider a variant of the example used in Reference [37] holding all standing assumptions stated in Section 2.2. The nominal system model ($G_{o}\left(q^{-1},\theta \right)$ and $H(q^{-1},\theta )$) corresponds to a Box-Jenkins model. For simplicity, we also consider that the error-model ($G_{\Delta }\left(q^{-1},\eta^{\left[r\right]}\right)$) corresponds to a second order FIR system in Equation (12) with an overlapped Gaussian mixture error-model distribution. We assume that there are not dynamic uncertainties at low frequencies, i.e., the error-model has zero static gain. In this case, we do not establish a comparison analysis with other approaches since the simulated data is generated using our proposed framework with SE and GMMs.

In contrast, we consider a second example to illustrate the flexibility of our proposed algorithm for modeling the error-model for the system in Equation (2). Assumption 4 is relaxed considering a model structure does not correspond to Equation (3). Instead, we consider the system in Equation (2) with an output-error model structure where the system model, $G^{\left[r\right]}\left(q^{-1},\theta \right)$, is given by the discrete-time model of a simple resistor-capacitor continuous-time system model. Here, we consider that the time constant τ is given by $\tau =\tau_{o}\left(1+\delta^{\left[r\right]}\right)$, where $\tau_{o}$ represents the time constant computed with the nominal values of the resistor and capacitor, and $\delta^{\left[r\right]}$ denotes the tolerance (uncertainty) with a non-Gaussian distribution.

For both examples, the simulation setup is as follows:

(1) The data length is N=100.
(2) The number of experiments is M=100.
(3) The number of Monte Carlo (MC) simulation is 25.
(4) The stopping criterion is satisfied when:
  $$\left\|{\hat{\beta }}^{\left(m\right)}-{\hat{\beta }}^{\left(m-1\right)}\right\|/\|{\hat{\beta }}^{\left(m\right)}\|<10^{-6},$$
  or when 2000 iterations of the EM algorithm have been reached, where ‖·‖ denotes the general vector norm operator.

Notice that each MC simulation corresponds to an estimation obtained using the data from *M* independent experiments.

On the other hand, for the numerical examples, the initialization of the estimation algorithm is as follows: For the nominal model initialization, we estimate a system model for each independent experiment using PEM and a sufficiently flexible (high order) structure. Then, we choose the estimated system model and the corresponding estimated noise variance with the smallest number of parameters that better described all the previously estimated models. For the error-model FIR transfer function, we adopt a similar approach for different FIR filter orders ($n_{\Delta }$). Here, we choose a sufficiently complex FIR system by assuming the nominal model is equal to one ($G_{o}\left(q^{-1},\theta \right)=1$). The number of Gaussian mixture model components (κ) is defined by the user, and it is typically a large number. Once the parameters have been estimated, it is possible to discard the Gaussian component corresponding to small estimated weight (e.g., 1/(100κ)). Finally, for a given κ, the initial distribution of the parameters of the error-model is chosen as follows:

(1) From all the estimated FIR models, compute the average value of the coefficient corresponding to each tap.
(2) Evenly space the initial mean values of the $n_{\Delta }$-dimensional GMM between the estimated maximum and the minimum value of each tap.
(3) Set the variances of the $n_{\Delta }$-dimensional GMM equal to a diagonal covariance matrix with the sample variance of each tap on the diagonal.
(4) Set the mixing weight for each GMM component equal to 1/κ.

### 5.1. Example 1: A General System with a Gaussian Mixture Error-Model Distribution

Consider the *true* system $G^{\left[r\right]}\left(q^{-1}\right)$ in Equation (2) with a nominal system model as follows:

(36) $$G_{o}\left(q^{-1},\theta \right)=\frac{b_{1}^{0}q^{-1}}{1+a_{1}^{0}q^{-1}},\;\;H\left(q^{-1},\theta \right)=\frac{1+c_{1}^{0}q^{-1}}{1+d_{1}^{0}q^{-1}},$$

and the error-model in Equation (12) is given by

(37) $$G_{\Delta }\left(q^{-1},\eta^{\left[r\right]}\right)=\eta_{0}^{\left[r\right]}+\eta_{1}^{\left[r\right]}q^{-1},$$

where $b_{1}^{0}=1$, $a_{1}^{0}=0.5$, $c_{1}^{0}=0.1$, and $d_{1}^{0}=0.8$. The input signal is $u_{t}^{\left[r\right]}∼N\left(0,\sigma_{u}^{2}\right)$, $\sigma_{u}^{2}=10$, and the noise source $\omega_{t}^{\left[r\right]}∼N\left(0,\sigma^{2}\right)$, $\sigma^{2}=0.1$. In this example, we focus on uncertainties in the system model at high frequencies, i.e., $\eta_{1}=-\eta_{0}$. We also consider that, for each experiment, $\eta^{\left[r\right]}={\left[\eta_{0}\;\eta_{1}\right]}^{T}$ is drawn from a Gaussian mixture distribution in Equation (4) with $\alpha_{1}=\alpha_{2}=0.5$, $\Gamma_{1}=\Gamma_{2}=0.2$, $\mu_{1}=-1$, and $\mu_{2}=1$. The vector of parameters to estimate is $\beta =\{\theta ,\gamma ,\sigma^{2}\}$ with $\theta =\{b_{1}^{0},a_{1}^{0},c_{1}^{0},d_{1}^{0}\}$ and $\gamma ={\left\{\alpha_{i},\mu_{i},\Gamma_{i}\right\}}_{i=1}^{\kappa }$ with κ=2. The initial values used for our proposed EM algorithm correspond to ${\hat{\theta }}^{\left(0\right)}={\left[1.2510\;0.4523\;0.0772\;0.7546\right]}^{T}$, ${\hat{\sigma }}^{2^{\left(0\right)}}=0.0612$, ${\hat{\alpha }}_{1}^{\left(0\right)}={\hat{\alpha }}_{2}^{\left(0\right)}=0.5$, ${\hat{\mu }}_{1}^{\left(0\right)}=-1.1962$, ${\hat{\mu }}_{2}^{\left(0\right)}=2.9673$, ${\hat{\Gamma }}_{1}^{\left(0\right)}={\hat{\Gamma }}_{2}^{\left(0\right)}=0.8651$.

**Figure 3:**
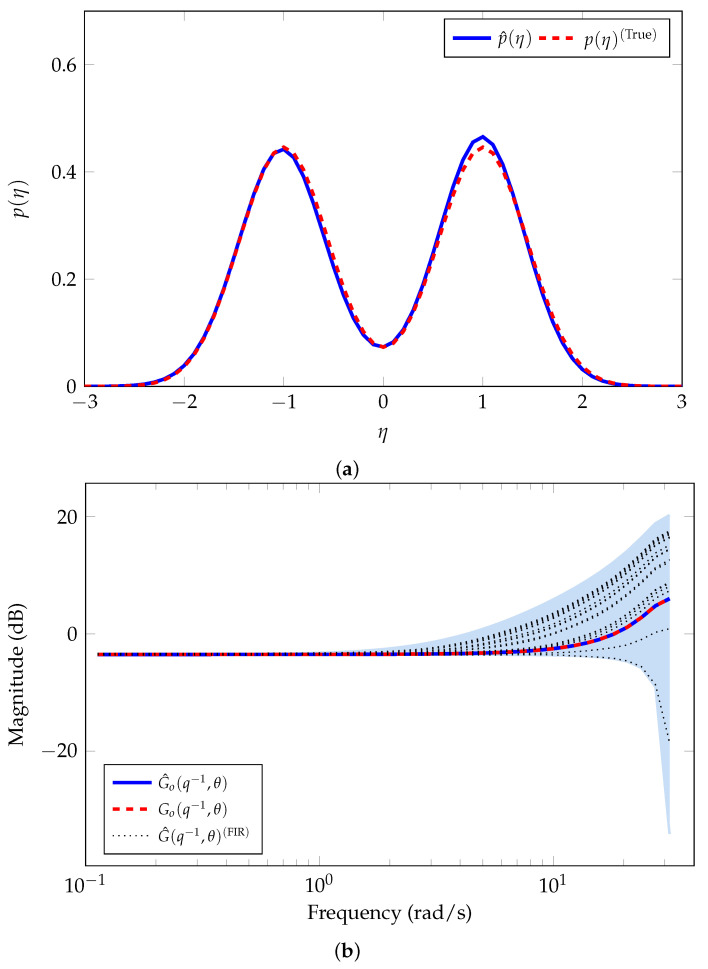
Nominal model and error-model distribution estimated for Example 1. (**a**) Estimated error-model distribution. (**b**) Estimated nominal model $G_{o}\left(q^{-1},\theta \right)$.

Table 1 shows the estimation results of the nominal model parameters and the noise variance. Figure 3a shows the results of the estimation of the error-model distribution. The blue line corresponds to the average of all the GMMs estimated. It is clear that the estimated PDF is similar to the *true* PDF.

On the other hand, we note that the SE method does not directly provide an uncertainty region. However, we compute *M* realizations of the model

(38) $${\hat{G}}_{o}\left(q^{-1},\hat{\theta }\right)+{\hat{G}}_{o}\left(q^{-1},\hat{\theta }\right)G_{\Delta }\left(q^{-1},\eta^{\left[r\right]}\right),$$

with r=1,…,M, where ${\hat{G}}_{o}\left(q^{-1},\hat{\theta }\right)$ is the nominal model estimated and $\eta^{\left[r\right]}$ are obtained from *M* different realizations using the GMM in Equation (4) with the estimated parameters $\hat{\gamma }$ obtained by using our proposed SE method. In addition, we use the PEM [1,2] to estimate the system model $G^{\left[r\right]}\left(q^{-1}\right)$ with a high order FIR model. We consider the measurements of 25 independent experiments, and we estimate a 10th order FIR system model, $\hat{G}{\left(q^{-1},\theta \right)}^{\left(\mathrm{FIR}\right)}$, for each independent experiment in order to validate the uncertainty region estimated.

Figure 3b shows the magnitude of the frequency response corresponding to the average of all MC simulations for the estimated nominal model (blue line). The red-dashed line corresponds to the nominal system model in Equation (36), i.e., $G_{o}\left(q^{-1},\theta \right)$. The blue-shaded region represents the area of the corresponding uncertainty region estimated of the *true* system. We observe an accurate estimation of the nominal system model. We also observe that the FIR models estimated (black-dotted lines) lie in the uncertainty region obtained with our proposed method.

### 5.2. Example 2: A Resistor-Capacitor System Model with Non-Gaussian-Sum Uncertainties

Consider the system in Equation (2) as follows:

(39) $$G^{\left[r\right]}\left(q^{-1},\tau^{\left[r\right]}\right)=\frac{q^{-1}\left(1-e^{-T_{s}/\tau^{\left[r\right]}}\right)}{1-\left(e^{-T_{s}/\tau^{\left[r\right]}}\right)q^{-1}},\;\;\;\;H\left(q^{-1}\right)=1,$$

where $T_{s}=0.01$ is the sampled period, and $\tau^{\left[r\right]}=\tau_{o}\left(1+\delta^{\left[r\right]}\right)$ is the time constant with $\tau_{o}=10\times 10^{-3}$, and $\delta^{\left[r\right]}$ is uniformly distributed as U[−0.4,0.4]. We consider that the sampled period $T_{s}$ is known.

For the SE method, we consider an output-error nominal model with a FIR error-model as follows:

(40) $$G_{o}\left(q^{-1},\theta \right)=\frac{q^{-1}\left(1-e^{-T_{s}/\tau_{o}}\right)}{1-\left(e^{-T_{s}/\tau_{o}}\right)q^{-1}},$$

(41) $$G_{\Delta }\left(q^{-1},\eta^{\left[r\right]}\right)=\eta_{0}-\eta_{0}q^{-1}.$$

The distribution of the parameters $\eta^{\left[r\right]}$ of the error-model is modeled using a GMM with $\gamma ={\left\{\alpha_{i},\mu_{i},\Gamma_{i}\right\}}_{i=1}^{\kappa }$, κ=2 components. The vector of parameter to be estimated is $\beta =\{\tau_{o},\gamma ,\sigma^{2}\}$. As in the previous example, the uncertainty region is obtained from Equation (38) with r=1,…,M, where $\eta^{\left[r\right]}$ is obtained from *M* different realizations using the GMM in Equation (4) with the estimated parameters $\hat{\gamma }$. In addition, the initial values used for our proposed EM algorithm are given by ${\hat{\tau }}_{0}^{\left(0\right)}=9\times 10^{-3}$, ${\hat{\sigma }}^{2^{\left(0\right)}}=0.1247$, ${\hat{\alpha }}_{1}^{\left(0\right)}={\hat{\alpha }}_{2}^{\left(0\right)}=0.5$, ${\hat{\mu }}_{1}^{\left(0\right)}=-0.0163$, ${\hat{\mu }}_{2}^{\left(0\right)}=0.8025$, ${\hat{\Gamma }}_{1}^{\left(0\right)}={\hat{\Gamma }}_{2}^{\left(0\right)}=0.1092$.

For comparison purposes, we estimate the system uncertainty and the nominal model using MEM approach [33]. The nominal model estimated, ${\hat{G}}_{o}{\left(q^{-1},\theta \right)}^{\left(\mathrm{MEM}\right)}$, corresponds to the average of all PEM estimations obtained using the original structure given in Equation (39), i.e., $n_{a}=n_{b}=1$ and $n_{c}=n_{d}=0$, from the all independent experiments. In order to obtain the residuals, we compute $\varepsilon_{t}=y_{t}-{\hat{G}}_{o}{\left(q^{-1},\theta \right)}^{\left(\mathrm{MEM}\right)}u_{t}$ for each independent experiment. The error-model, $G_{\varepsilon }\left(q^{-1},\theta_{\varepsilon }\right)$, is obtained from the residual data as follows:

(42) $$\varepsilon_{t}=G_{\varepsilon }\left(q^{-1},\theta_{\varepsilon }\right)u_{t}+v_{t},$$

where $v_{t}$ is a zero-mean Gaussian noise sequence which is assumed to be uncorrelated with the input signal $u_{t}$. Thus, we consider a 10th order FIR system model for $G_{\varepsilon }\left(q^{-1},\theta_{\varepsilon }\right)$ and we use the PEM [2] to estimate the error-model parameters $\theta_{\varepsilon }$ for each experiment. The uncertainty region is computed by adding frequency by frequency the average of the all error-model estimated to the nominal model.

Finally, we consider the measurements of 25 independent experiments and we obtain an estimation of the system model in Equation (39), $\hat{G}{\left(q^{-1},\theta \right)}^{\left(\mathrm{PEM}\right)}$, using PEM with $n_{a}=n_{b}=1$ and $n_{c}=n_{d}=0$ for each independent experiment in order to validate the uncertainty region estimated for both SE and MEM approach.

**Figure 4:**
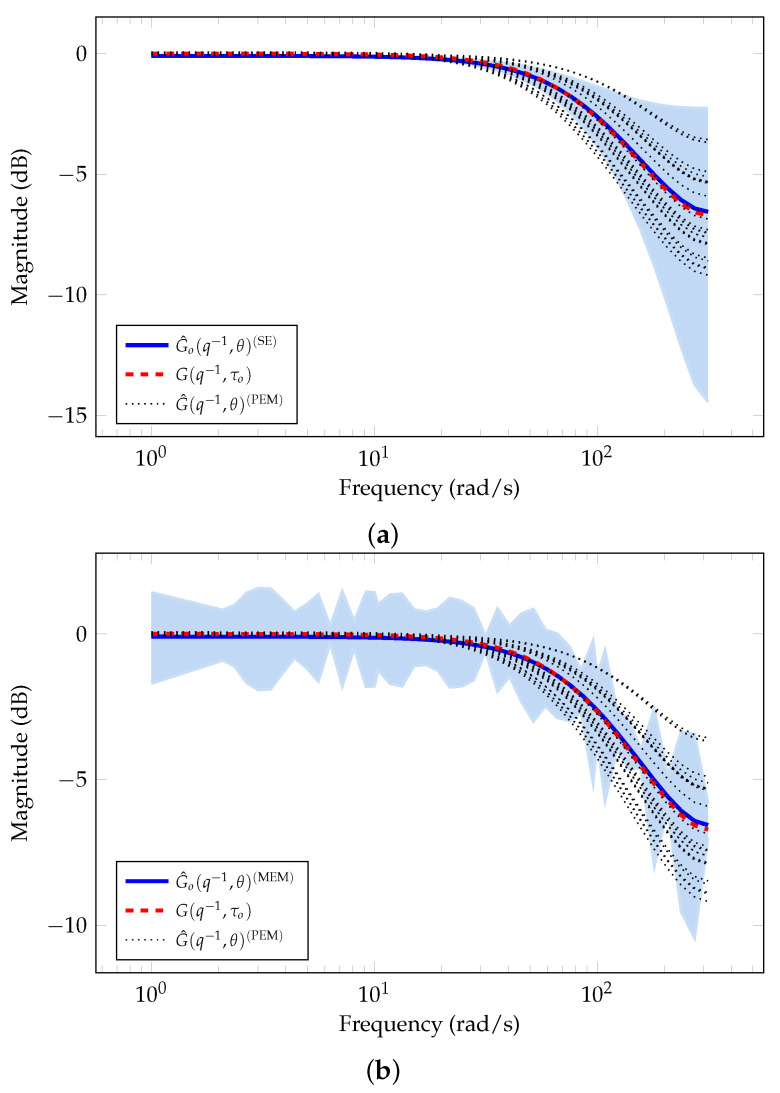
Nominal model and uncertainty region estimated using SE and MEM for Example 2. (**a**) System model estimated with SE. (**b**) System model estimated with MEM.

Figure 4a shows the magnitude of the frequency response corresponding to the nominal model and the uncertainty region estimated using SE approach. The red-dashed line corresponds to the system model in Equation (39) considering the time constant without uncertainty, i.e., $G(q^{-1},\tau_{o})$. The blue line corresponds to the average of all MC simulations for the estimated nominal model. We observe an accurate estimation for the nominal model with ${\hat{\tau }}_{o}=9.7\times 10^{-3}\pm 0.17\times 10^{-3}$ and noise variance ${\hat{\sigma }}^{2}=0.133\pm 5.9\times 10^{-3}$. The blue-shaded area corresponds to the uncertainty region estimated for the *true* system. Similarly, Figure 4b shows the magnitude of the frequency response corresponding to the nominal model estimated (blue line) and the uncertainty region estimated (blue-shaded area) from the residuals using MEM. We also observe an accurate estimation of the nominal model with ${\hat{\tau }}_{o}=10.1\times 10^{-3}\pm 2.30\times 10^{-3}$ and noise variance ${\hat{\sigma }}^{2}=0.099\pm 2.85\times 10^{-3}$. In addition, we observe in Figure 4a,b that PEM estimations, $\hat{G}{\left(q^{-1},\theta \right)}^{\left(\mathrm{PEM}\right)}$, lie in the uncertainty region obtained using both SE approach and MEM. However, SE method describes better the uncertainty region than MEM method, specifically, the error-model at low frequencies.

## 6. Conclusions

In this paper, we have addressed the uncertainty modeling problem by combining the SE approach with GMMs. We proposed an identification algorithm using ML principle to estimate both the nominal model and the distribution of the parameters of the error-model as a GMM. An EM algorithm is proposed to solve the ML estimation problem providing closed-form expressions for the estimators of the GMM parameters and the noise variance. In the simulation examples, we considered two scenarios: (i) identification of a system model with a structure defined with SE approach using a nominal model and an error-model as a FIR system with Gaussian mixture distribution, and (ii) identification of a system model with non-Gaussian uncertainties in the parameters does not correspond to the SE framework. For both cases, we obtained accurate estimations of the nominal system model. Finally, our proposed method provided a good description of the uncertainty region in the system for the simulation examples.

## Figures and Tables

**Table 1:** Nominal model parameters and noise variance estimated for Example 1.

Parameter	True Value	Estimated Value
$b_{1}$	1	$0.9994\pm 1.54\times 10^{-3}$
$a_{1}$	0.5	$0.4998\pm 4.53\times 10^{-4}$
$c_{1}$	0.1	$0.0997\pm 1.17\times 10^{-2}$
$d_{1}$	0.8	$0.8017\pm 5.97\times 10^{-3}$
$\sigma^{2}$	0.1	$0.1003\pm 1.44\times 10^{-3}$

## Data Availability

No new data were created or analyzed in this study. Data sharing is not applicable to this article.

## Abbreviations

The following abbreviations are used in this manuscript:

EM:	Expectation-Maximization
FIR:	Finite impulse response
GMM:	Gaussian mixture model
MC:	Monte Carlo
MEM:	Model error modeling
ML:	Maximum likelihood
PDF:	Probability density function
PEM:	Prediction error method
SE:	Stochastic embedding

## Appendix A. Maximum Likelihood Formulation with SE Approach

Let us consider that $Y^{\left[r\right]}=\left[y_{1}^{\left[r\right]}\;\cdots \;y_{N}^{\left[r\right]}\right]$ is a collection of measurements for each experiment r=1,…,M. Then, the likelihood function, L(β), for the system model in Equation (13) using the GMM in Equation (4) can be obtained by marginalizing the hidden variable, $\left[\eta^{\left[1\right]}\;\cdots \;\eta^{\left[M\right]}\right]$, as follows:

(A1) $$\begin{array}{cc} L(\beta ) & =p(Y^{\left[1\right]},\ldots ,Y^{\left[M\right]}|\beta )\\ \; & =\prod_{r=1}^{M}\int_{-\infty }^{\infty }p\left(Y^{\left[r\right]}|\eta^{\left[r\right]},\beta \right)p\left(\eta^{\left[r\right]}|\beta \right)d\eta^{\left[r\right]}, \end{array}$$

where

(A2) $$p\left(Y^{\left[r\right]}|\eta^{\left[r\right]},\beta \right)=N\left(Y^{\left[r\right]};G\left(\theta \right)U^{\left[r\right]}+\Psi^{\left[r\right]}\left(\theta \right)\eta^{\left[r\right]},\sigma^{2}I_{N}\right),$$

(A3) $$p\left(\eta^{\left[r\right]}|\beta \right)=\sum_{i=1}^{\kappa }\alpha_{i}N\left(\eta^{\left[r\right]};\mu_{i},\Gamma_{i}\right),$$

with $\alpha_{i}>0$, $\sum_{i=1}^{\kappa }\alpha_{i}=1$. The log-likelihood function, ℓ(β), is then given by

(A4) $$\ell \left(\beta \right)=\sum_{r=1}^{M}\log\left\{\sum_{i=1}^{\kappa }\alpha_{i}\int_{-\infty }^{\infty }p\left(Y^{\left[r\right]}|\eta^{\left[r\right]},\beta \right)p\left(\eta^{\left[r\right]}|\beta \right)d\eta^{\left[r\right]}\right\}.$$

Consider the following identities:

(A5) $$\;\left[\begin{array}{cc} A & \;B\\ C & \;D \end{array}\right]=\left[\begin{array}{cc} I & \;0\\ CA^{-1} & \;I \end{array}\right]\;\left[\begin{array}{cc} A & \;0\\ 0 & \;D-CA^{-1}B \end{array}\right]\;\left[\begin{array}{cc} I & \;A^{-1}B\\ 0 & \;I \end{array}\right],$$

(A6) $${\left(I+BD^{-1}C\right)}^{-1}=I-B{\left(D+CB\right)}^{-1}C,$$

(A7) $$\;\det\left(A\right)\det\left(D-CA^{-1}B\right)=\det\left(D\right)\det\left(A-BD^{-1}C\right).$$

From Equation (A4), we define $K_{i}\left(\beta ,\eta^{\left[r\right]}\right)=p\left(Y^{\left[r\right]}|\eta^{\left[r\right]},\beta \right)p\left(\eta^{\left[r\right]}|\beta \right)$. Substituting Equations (A2) and (A3) in $K_{i}\left(\beta ,\eta^{\left[r\right]}\right)$, and using the identities in Equations (A5)–(A7), we obtain:

(A8) $$K_{i}\left(\beta ,\eta^{\left[r\right]}\right)=N\left(Y^{\left[r\right]};{\bar{\mu }}_{ir},{\bar{\Sigma }}_{ir}\right)N\left(\eta^{\left[r\right]};{\tilde{\mu }}_{ir},{\tilde{\Sigma }}_{ir}\right),$$

with

(A9) $${\bar{\mu }}_{ir}=\Psi^{\left[r\right]}\left(\theta \right)\mu_{i}+G\left(\theta \right)U^{\left[r\right]},$$

(A10) $${\bar{\Sigma }}_{ir}=\sigma^{2}I_{N}+\Psi^{\left[r\right]}\left(\theta \right)\Gamma_{i}{\left[\Psi^{\left[r\right]}\left(\theta \right)\right]}^{T},$$

(A11) $$P_{ir}=\Gamma_{i}{\left[\Psi^{\left[r\right]}\left(\theta \right)\right]}^{T}{\left({\bar{\Sigma }}_{ir}\right)}^{-1},$$

(A12) $${\tilde{\mu }}_{ir}=\mu_{i}+P_{ir}\left(Y^{\left[r\right]}-{\bar{\mu }}_{ir}\right),$$

(A13) $${\tilde{\Sigma }}_{ir}=\left(I_{n_{\Delta }}-P_{ir}\Psi^{\left[r\right]}\left(\theta \right)\right)\Gamma_{i}.$$

Substituting Equation (A8) in Equation (A4), we can directly obtain the log-likelihood function in Equation (16). Then, the ML estimator corresponds to Equation (15).

## Appendix B. Computing the Complete Likelihood Function

From the likelihood function in Equation (16), we have:

(A14) $$p\left(Y^{\left[r\right]}|\beta \right)=\sum_{i=1}^{\kappa }\alpha_{i}N\left(Y^{\left[r\right]};{\bar{\mu }}_{ir},{\bar{\Sigma }}_{ir}\right),$$

where ${\bar{\mu }}_{ir}$ and ${\bar{\Sigma }}_{ir}$ are given by Equations (A9) and (A10), respectively. Let consider

(A15) $$p\left(\zeta_{r}=i\right)=\alpha_{i},\;\;\;i=1,\ldots ,\kappa ,$$

where $\zeta_{1:M}$ is a discrete random variable (hidden variable) such that $\zeta_{r}$ is an indicator that determines if the *r*th observation $Y^{\left[r\right]}$ arises from the *i*th component of the GMM in Equation (A3), that is,

(A16) $$\zeta_{r}\in \left\{1,\ldots ,\kappa \right\},\;\;r=1,\ldots ,M.$$

Then, Equation (A14) can be obtained by marginalizing the hidden variable, $\zeta_{r}$, as follows:

(A17) $$p\left(Y^{\left[r\right]}|\beta \right)=\sum_{i=1}^{\kappa }p\left(Y^{\left[r\right]}|\zeta_{r}=i,\beta \right)p\left(\zeta_{r}=i|\beta \right),$$

where

(A18) $$p\left(Y^{\left[r\right]}|\zeta_{r}=i,\beta \right)=N\left(Y^{\left[r\right]};{\bar{\mu }}_{ir},{\bar{\Sigma }}_{ir}\right).$$

Defining the complete data set as $\left\{Y^{\left[1:M\right]},\zeta_{1:M}\right\}$ and utilizing the Bayes’ rule, the complete likelihood function is obtained as follows:

(A19) $$p\left(Y^{\left[1:M\right]},\zeta_{1:M}|\beta \right)=\prod_{r=1}^{M}p\left(Y^{\left[r\right]}|\zeta_{r},\beta \right)p\left(\zeta_{r}|\beta \right).$$

The complete log-likelihood function is given by:

(A20) $$\begin{array}{c} \log\left[p(Y^{\left[1:M\right]},\zeta_{1:M}|\beta )\right]=\sum_{r=1}^{M}\log\left[p\left(\zeta_{r}|\beta \right)\right]+\log\left[p\left(Y^{\left[r\right]}|\zeta_{r},\beta \right)\right]. \end{array}$$

Using Equations (A16) and (A17) in Equation (A20), we directly obtain Equation (21).

## Appendix C. EM Algorithm with GMMs Formulation

### Appendix C.1. Computing the Auxiliary Function $Q(\beta ,{\hat{\beta }}^{\left(m\right)})$

Using Equation (21) in Equation (19), we have:

(A21) $$\begin{array}{cc} Q(\beta ,{\hat{\beta }}^{\left(m\right)}) & =\sum_{r=1}^{M}\sum_{i=1}^{\kappa }E\left\{\log\left[p\left(\zeta_{r}=i|\beta \right)\right]|Y^{\left[1:M\right]},{\hat{\beta }}^{\left(m\right)}\right\}\\ \; & +E\left\{\log\left[N\left(Y^{\left[r\right]};{\bar{\mu }}_{ir},{\bar{\Sigma }}_{ir}\right)\right]|Y^{\left[1:M\right]},{\hat{\beta }}^{\left(m\right)}\right\}. \end{array}$$

Solving the expected value in Equation (A21) and using Equation (A15) into Equation (A21), we have:

(A22) $$\begin{array}{c} Q\left(\beta ,{\hat{\beta }}^{\left(m\right)}\right)=\sum_{r=1}^{M}\sum_{i=1}^{\kappa }\log\left[\alpha_{i}\right]{\hat{\zeta }}_{ir}^{\left(m\right)}+\log\left[N\left(Y^{\left[r\right]};{\bar{\mu }}_{ir},{\bar{\Sigma }}_{ir}\right)\right]{\hat{\zeta }}_{ir}^{\left(m\right)}, \end{array}$$

where

(A23) $${\hat{\zeta }}_{ir}^{\left(m\right)}=p\left(\zeta_{r}=i|Y^{\left[1:M\right]},{\hat{\beta }}^{\left(m\right)}\right).$$

Using the Bayes’ Theorem, Equations (A14), (A15), and (A17) to solve Equation (A23), we obtain:

(A24) $$\begin{array}{cc} {\hat{\zeta }}_{ir}^{\left(m\right)} & =\frac{p\left(\zeta_{r}=i|{\hat{\beta }}^{\left(m\right)}\right)p\left(Y^{\left[r\right]}|\zeta_{r}=i,{\hat{\beta }}^{\left(m\right)}\right)}{p(Y^{\left[r\right]}|{\hat{\beta }}^{\left(m\right)})}, \end{array}$$

(A25) $$\begin{array}{cc} \; & =\frac{{\hat{\alpha }}_{i}^{\left(m\right)}N\left(Y^{\left[r\right]};{\hat{\bar{\mu }}}_{ir}^{\left(m\right)},{\hat{\bar{\Sigma }}}_{ir}^{\left(m\right)}\right)}{\sum_{l=1}^{\kappa }{\hat{\alpha }}_{l}^{\left(m\right)}N\left(Y^{\left[r\right]};{\hat{\bar{\mu }}}_{lr}^{\left(m\right)},{\hat{\bar{\Sigma }}}_{lr}^{\left(m\right)}\right)}, \end{array}$$

where ${\hat{\bar{\mu }}}_{ir}^{\left(m\right)}$ and ${\hat{\bar{\Sigma }}}_{ir}^{\left(m\right)}$ are given by Equations (24) and (25), respectively.

### Appendix C.2. Optimizing the Auxiliary Function $Q(\beta ,{\hat{\beta }}^{\left(m\right)})$

From Equation (A8), we have the following:

(A26) $$\int_{-\infty }^{\infty }\log\left[K_{i}\left(\beta ,\eta^{\left[r\right]}\right)\right]d\eta^{\left[r\right]}=\log\left[N(Y^{\left[r\right]};{\bar{\mu }}_{ir},{\bar{\Sigma }}_{ir})\right].$$

Let consider the following identity for a random variable *x*:

(A27) $$E\left\{{\left(b-Ax\right)}^{T}\left(b-Ax\right)\right\}={\left(b-A\hat{x}\right)}^{T}\left(b-A\hat{x}\right)+\mathrm{tr}\left\{A\Sigma A^{T}\right\},$$

where $\hat{x}=E\left\{x\right\}$, and $\Sigma =E\left\{\left(x-\hat{x}\right){\left(x-\hat{x}\right)}^{T}\right\}$.

Using Equation (A27) to compute $\log\left[K_{i}\left(\beta ,\eta^{\left[r\right]}\right)\right]$ in Equation (A26), we obtain:

(A28) $$\begin{array}{cc} \; & \log\left[K_{i}\left(\beta ,\eta^{\left[r\right]}\right)\right]=\frac{N}{2}\log\left[\sigma^{2}\right]-\frac{1}{2}\log\left[\det\left(\Gamma_{i}\right)\right]-\frac{1}{2\sigma^{2}}\left[{\left(Y^{\left[r\right]}-G\left(\theta \right)U^{\left[r\right]}-\Psi^{\left[r\right]}\left(\theta \right){\hat{\tilde{\mu }}}_{ir}^{\left(m\right)}\right)}^{T}\right.\\ \; & \left.\left(Y^{\left[r\right]}-G\left(\theta \right)U^{\left[r\right]}-\Psi^{\left[r\right]}\left(\theta \right){\hat{\tilde{\mu }}}_{ir}^{\left(m\right)}\right)+\mathrm{tr}\left(\Psi^{\left[r\right]}\left(\theta \right){\hat{\tilde{\Sigma }}}_{ir}^{\left(m\right)}{\left[\Psi^{\left[r\right]}\left(\theta \right)\right]}^{T}\right)\right]-\frac{1}{2}\left[\mathrm{tr}(\Gamma_{i}^{-1}{\hat{\tilde{\Sigma }}}_{ir}^{\left(m\right)})+\right.\\ \; & \left.{\left({\hat{\tilde{\mu }}}_{ir}^{\left(m\right)}-\mu_{i}\right)}^{T}\Gamma_{i}^{-1}\left({\hat{\tilde{\mu }}}_{ir}^{\left(m\right)}-\mu_{i}\right)\right]. \end{array}$$

Substituting Equations (A26) and (A28) in Equation (A22), and taking the derivative of Equation (A22) with respect to $\mu_{i}$ and equating to zero, yields:

(A29) $$\frac{\partial Q(\beta ,{\hat{\beta }}^{\left(m\right)})}{\partial \mu_{i}}=\sum_{r=1}^{M}\left({\hat{\tilde{\mu }}}_{ir}^{\left(m\right)}-{\hat{\mu }}_{i}^{\left(m+1\right)}\right){\hat{\zeta }}_{ir}^{\left(m\right)}=0.$$

From Equation (A29), we directly obtain Equation (27).

Then, taking the derivative of Equation (A22) with respect to $\Gamma_{i}^{-1}$ and equating to zero:

(A30) $$\begin{array}{c} \frac{\partial Q(\beta ,{\hat{\beta }}^{\left(m\right)})}{\partial \Gamma_{i}^{-1}}=\sum_{r=1}^{M}\left(\left[\left({\hat{\tilde{\mu }}}_{ir}^{\left(m\right)}-{\hat{\mu }}_{i}^{\left(m\right)}\right){\left({\hat{\tilde{\mu }}}_{ir}^{\left(m\right)}-{\hat{\mu }}_{i}^{\left(m\right)}\right)}^{T}+{\hat{\tilde{\Sigma }}}_{ir}^{\left(m\right)}\right]-{\hat{\Gamma }}_{i}^{\left(m+1\right)}\right){\hat{\zeta }}_{ir}^{\left(m\right)}=0. \end{array}$$

From Equation (A30), we can directly obtain Equation (28).

Then, taking the derivative of Equation (A22) with respect to $\rho =1/\sigma^{2}$ and equating to zero yields:

(A31) $$\begin{array}{cc} \frac{1}{N}\sum_{r=1}^{M}\sum_{i=1}^{\kappa } & \left[{\left(Y^{\left[r\right]}-G\left({\hat{\theta }}^{\left(m\right)}\right)U^{\left[r\right]}-\Psi^{\left[r\right]}\left({\hat{\theta }}^{\left(m\right)}\right){\hat{\tilde{\mu }}}_{ir}^{\left(m\right)}\right)}^{T}\left(Y^{\left[r\right]}-G\left({\hat{\theta }}^{\left(m\right)}\right)U^{\left[r\right]}-\Psi^{\left[r\right]}\left({\hat{\theta }}^{\left(m\right)}\right){\hat{\tilde{\mu }}}_{ir}^{\left(m\right)}\right)+\right.\\ \; & \left.\mathrm{tr}\left(\Psi^{\left[r\right]}\left({\hat{\theta }}^{\left(m\right)}\right){\hat{\tilde{\Sigma }}}_{ir}^{\left(m\right)}{\left[\Psi^{\left[r\right]}\left({\hat{\theta }}^{\left(m\right)}\right)\right]}^{T}\right)\right]{\hat{\zeta }}_{ir}^{\left(m\right)}=\sum_{r=1}^{M}\sum_{i=1}^{\kappa }{\hat{\rho }}^{\left(m+1\right)}{\hat{\zeta }}_{ir}^{\left(m\right)}. \end{array}$$

Using ${\hat{\sigma }}^{2^{\left(m+1\right)}}=1/{\hat{\rho }}^{\left(m+1\right)}$ and Equation (33) in Equation (A31), we directly obtain Equation (29).

For the parameter $\alpha_{i}$, we define $F(\alpha_{i})$ as follows:

(A32) $$F\left(\alpha_{i}\right)=\sum_{r=1}^{M}\sum_{i=1}^{\kappa }\log\left[\alpha_{i}\right]{\hat{\zeta }}_{ir}^{\left(m\right)},$$

subject to $\sum_{i=1}^{\kappa }\alpha_{i}=1$. Then, using a Lagrange multiplier to deal with the constraint on $\alpha_{i}$, we define:

(A33) $$J\left(\alpha_{i},\nu \right)=\sum_{r=1}^{M}\sum_{i=1}^{\kappa }\log\left[\alpha_{i}\right]{\hat{\zeta }}_{ir}^{\left(m\right)}+\nu \left(\sum_{i=1}^{\kappa }\alpha_{i}-1\right).$$

Taking the derivative of Equation (A33) with respect to $\alpha_{i}$ and ν and equating to zero, we obtain:

(A34) $$\frac{\partial J(\alpha_{i},\nu )}{\partial \alpha_{i}}=\sum_{r=1}^{M}\frac{{\hat{\zeta }}_{ir}^{\left(m\right)}}{{\hat{\alpha }}_{i}^{\left(m+1\right)}}-\nu =0,$$

(A35) $$\frac{\partial J(\alpha_{i},\nu )}{\partial \nu }=\sum_{i=1}^{\kappa }\alpha_{i}-1=0.$$

Then, ${\hat{\alpha }}_{i}^{\left(m+1\right)}=\sum_{r=1}^{M}{\hat{\zeta }}_{ir}^{\left(m\right)}/\nu$. Taking summation over i=1,...,κ and using Equation (A35), we have:

(A36) $$\sum_{i=1}^{\kappa }{\hat{\alpha }}_{i}^{\left(m+1\right)}=\sum_{i=1}^{\kappa }\sum_{r=1}^{M}{\hat{\zeta }}_{ir}^{\left(m\right)}/\nu ,$$

(A37) $$\nu =\sum_{i=1}^{\kappa }\sum_{r=1}^{M}{\hat{\zeta }}_{ir}^{\left(m\right)}.$$

Notice that $\sum_{i=1}^{\kappa }{\hat{\zeta }}_{ir}^{\left(m\right)}=1$, and then we have ν=M. Then, we can obtain Equation (26). Notice that $0\leq {\hat{\alpha }}_{i}^{\left(m+1\right)}\leq 1$ holds even though we did not explicitly consider it in Equation (A33).

Finally, substituting Equations (A26) and (A28) in Equation (A22), we can directly obtain Equation (35) for the parameter θ.
